# The Neurotranscriptome of *Monochamus alternatus*

**DOI:** 10.3390/ijms25084553

**Published:** 2024-04-22

**Authors:** Xiaohong Han, Mingqing Weng, Wenchao Shi, Yingxin Wen, Yirong Long, Xinran Hu, Guoxi Ji, Yukun Zhu, Xuanye Wen, Feiping Zhang, Songqing Wu

**Affiliations:** 1 College of Forestry, Fujian Agriculture and Forestry University, Fuzhou 350002, China; hxhdax@163.com (X.H.); 3200422071@fafu.edu.cn (M.W.); 13950614441@163.com (W.S.); 18050409132@139.com (Y.W.); lyr-04128@foxmail.com (Y.L.); 18208368769@163.com (X.H.); jgxybyqkkk@163.com (G.J.); zhuyukun001@126.com (Y.Z.); wenxuanye_1116@163.com (X.W.); 2 Key Laboratory of Integrated Pest Management in Ecological Forests, Fujian Province University, Fujian Agriculture and Forestry University, Fuzhou 350002, China; 3 Fujian Colleges and Universities Engineering Research Institute of Conservation and Utilization of Natural Bioresources, Fujian Agriculture and Forestry University, Fuzhou 350002, China

**Keywords:** pine wilt disease, *Monochamus alternatus*, olfactory recognition protein, mRNA sequencing, de novo genome assembly, chemosensory receptors

## Abstract

The Japanese pine sawyer *Monochamus alternatus* serves as the primary vector for pine wilt disease, a devastating pine disease that poses a significant threat to the sustainable development of forestry in the Eurasian region. Currently, trap devices based on informational compounds have played a crucial role in monitoring and controlling the *M. alternatus* population. However, the specific proteins within *M. alternatus* involved in recognizing the aforementioned informational compounds remain largely unclear. To elucidate the spatiotemporal distribution of *M. alternatus* chemosensory-related genes, this study conducted neural transcriptome analyses to investigate gene expression patterns in different body parts during the feeding and mating stages of both male and female beetles. The results revealed that 15 genes in the gustatory receptor (GR) gene family exhibited high expression in the mouthparts, most genes in the odorant binding protein (OBP) gene family exhibited high expression across all body parts, 22 genes in the odorant receptor (OR) gene family exhibited high expression in the antennae, a significant number of genes in the chemosensory protein (CSP) and sensory neuron membrane protein (SNMP) gene families exhibited high expression in both the mouthparts and antennae, and 30 genes in the ionotropic receptors (IR) gene family were expressed in the antennae. Through co-expression analyses, it was observed that 34 genes in the IR gene family were co-expressed across the four developmental stages. The Antenna IR subfamily and IR8a/Ir25a subfamily exhibited relatively high expression levels in the antennae, while the Kainate subfamily, NMDA subfamily, and Divergent subfamily exhibited predominantly high expression in the facial region. *MalIR33* is expressed only during the feeding stage of *M. alternatus*, the *MalIR37* gene exhibits specific expression in male beetles, the *MalIR34* gene exhibits specific expression during the feeding stage in male beetles, the *MalIR8* and *MalIR39* genes exhibit specific expression during the feeding stage in female beetles, and *MalIR8* is expressed only during two developmental stages in male beetles and during the mating stage in female beetles. The IR gene family exhibits gene-specific expression in different spatiotemporal contexts, laying the foundation for the subsequent selection of functional genes and facilitating the full utilization of host plant volatiles and insect sex pheromones, thereby enabling the development of more efficient attractants.

## 1. Introduction

*Monochamus alternatus*, also known as the Japanese pine sawyer beetle, is a significant global pine tree pest, and it is also the primary vector responsible for transmitting the devastating pine wilt disease caused by the pine wood nematode [1,2,3,4]. Since the discovery of this disease on *Pinus thunbergii* in Zhongshan Mausoleum in Nanjing, Jiangsu Province, China in 1982, over the past few decades, the epidemic area has expanded to include 731 county-level administrative regions in 19 provinces and municipalities, including Fujian, Sichuan, and Hunan. Currently, the disease has been reported in multiple countries, including the United States, Japan, South Korea, Vietnam, and China [5,6,7,8]. The disease has caused severe damage to China’s forest resources and ecological environment, leading to a continuous reduction in the area of dominant tree species, especially the pioneering reforestation species of *Pinus massoniana*. The disease not only causes significant economic losses in China but also poses a serious threat to the country’s forest security [9,10]. Therefore, the urgency of controlling and preventing pine wilt disease cannot be overstated. *M. alternatus* serves as the key vector for the transmission of pine wilt disease, and the most effective measure in combating the disease is to suppress the spread of the Japanese pine sawyer beetle.

Existing research studies have identified several methods for controlling the Japanese pine sawyer beetle, including predictive forecasting, physical control, chemical control, silvicultural control, and biological control [11,12]. The combination of chemical ecology and behavioral studies is characterized by its ability to develop highly efficient, low-toxicity, and widely applicable solutions, making it a widely utilized approach in both research and practical production. Meanwhile, chemical ecology has transitioned from investigating the relationship between insects and the environment to permeating various disciplines. Exploring plant secondary metabolites, chemical ecology has evolved into an important approach that can potentially uncover new avenues for controlling *M. alternatus* [13]. Pheromone compounds are a crucial category of chemical signals in chemical control measures, playing a significant role in the control of *M. alternatus* [14]. These can be broadly categorized into two types: volatile substances released by the host, such as α-Pinene and β-Pinene, and insect-secreted pheromones, including aggregation pheromones and sex pheromones [15,16]. The volatile compounds released by hosts can assist *M. alternatus* in host recognition, foraging, and oviposition site selection behaviors, while the insect’s self-secreted pheromones can induce certain physiological effects in *M. alternatus*, such as mate-seeking and mating behaviors, subsequently affecting oviposition and egg-laying activities [15].

Current research indicates that the combined use of host plant volatiles and insect sex pheromones can significantly enhance the trapping efficiency of traps. Traps developed based on this principle have been widely promoted in over 20 provinces in mainland China, and they are implemented in provinces affected by pine wilt disease outbreaks, effectively containing the spread of the disease [14,17]. As an efficient, non-resistant, highly specific, and non-polluting control method, the integration of traps with the behavior of *M. alternatus* and the comprehensive management of harmful organisms comprise a promising approach. By combining pheromone monitoring in chemical control with physical control, there is a broad scope for effectively managing *M. alternatus* populations. However, the receptors related to the target chemical information substances of *M. alternatus* are not yet fully understood. Therefore, gaining a deeper understanding of the identification mechanism of *M. alternatus* chemosensory-related genes and elucidating their functions are essential for enhancing the effectiveness of comprehensive control measures against *M. alternatus*. This is crucial in slowing down or preventing the evolution of resistant populations. Additionally, it lays the theoretical foundation for the development of more efficient, safer, and more specific attractants and repellents.

## 2. Results

### 2.1. The Analysis of the Spatiotemporal Expression Levels of Hte CSP Genes of M. alternatus

**Figure 1:**
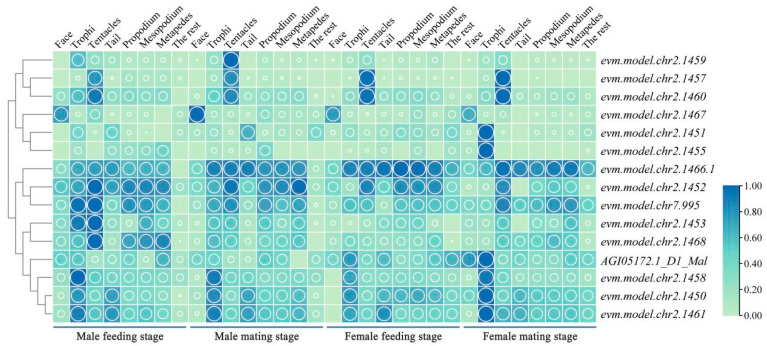
Spatiotemporal expression heatmap of CSP genes in *M. alternatus*. Green represents the low expression level of CSP genes in various tissues of *M. alternatus*, blue represents the high expression level of CSP genes in various tissues of *M. alternatus*. The larger the circle, the higher the expression level.

The spatiotemporal expression profiles of 15 CSP genes in the Japanese pine sawyer beetle were established based on the beetle’s genomic data, considering different genders, stages, and body parts. A heatmap depicting this expression pattern is shown in Figure 1. The results indicated that the expression of CSP genes was predominantly concentrated in the antennae and mouthparts. During the feeding stage of males, there are five highly expressed genes in the antennae and four highly expressed genes in the mouthparts. And during the mating stage of males, there are six and four highly expressed genes in the antennae and mouthparts, respectively. However, during the feeding stage of females, there are four highly expressed genes in the antennae and one highly expressed gene in the mouthparts. In contrast, during the mating stage of females, there are five highly expressed genes in the antennae and six highly expressed genes in the mouthparts. The *evm.TU.chr*2.1466.1 exhibited high expression in all stages, while *evm.TU.chr*2.1457 and *evm.TU.chr*2.1460 exhibited high expression in the antennae of both male and female beetles. The *evm.TU.chr*2.1459 demonstrated high expression in the antennae of male beetles during the mating stage, and *evm.TU.chr*2.1467 exhibited high expression in the facial region of both male and female beetles across all stages. Additionally, *AG105172.1*-D1, *evm.TU.chr*2.1451, *evm.TU.chr*2.1458, *evm.TU.chr*2.1450, *evm.TU.chr*2.1461, and *evm.TU.chr*2.1455 exhibited high expression in the mouthparts.

### 2.2. The Analysis of the Spatiotemporal Expression Levels of the SNMP Genes of M. alternatus

**Figure 2:**
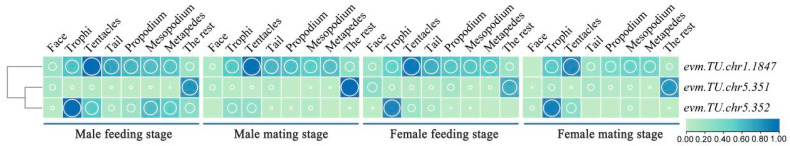
Spatiotemporal expression heatmap of SNMP genes in *M. alternatus*. Green represents the low expression level of SNMP genes in various tissues of *M. alternatus*, blue represents the high expression level of SNMP genes in various tissues of *M. alternatus*. The larger the circle, the higher the expression level.

The analysis of three SNMP genes in the transcriptome data of *M. alternatus* was performed. A heatmap depicting this expression pattern is shown in Figure 2. The results indicated that the expression levels of SNMP genes do not show significant differences between male and female beetles. However, there is specifically high expression in the mouthparts, antennae, and other body parts. Specifically, *evm.TU.chr*1.1847 exhibited high expression in other body parts, *evm.TU.chr*5.351 demonstrated high expression in the mouthparts, and *evm.TU.chr*5.352 exhibited high expression in the antennae.

### 2.3. The Analysis of the Spatiotemporal Expression Levels of the OBP Genes of M. alternatus

**Figure 3:**
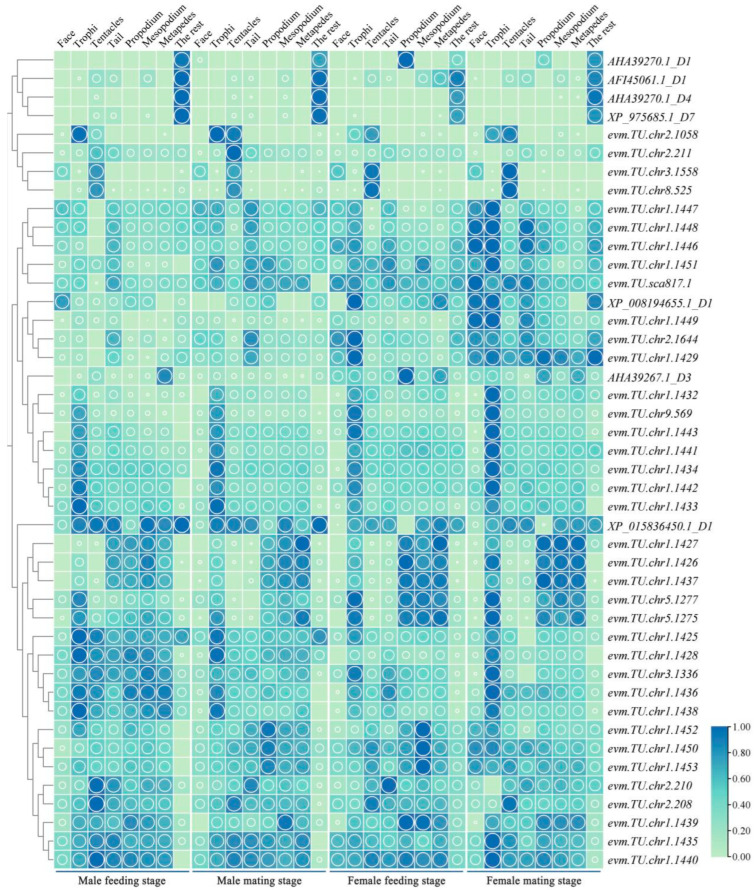
Heatmap of OBP genes expression in different parts of *M. alternatus*. Green represents the low expression level of OBP genes in various tissues of *M. alternatus*, blue represents the high expression level of OBP genes in various tissues of *M. alternatus*. The larger the circle, the higher the expression level.

We analyzed 44 OBP genes, as shown in Figure 3. As depicted in Figure 3, 34 OBP genes were expressed in the mouthparts and facial region of female beetles, with 34 genes co-expressed in the fore-, middle, and hind legs. Among these, *AHA39270.1*_D1, *AF145061.1*_D1, *AHA39270.1*_D4, and *XP_975685.1*_D7 exhibited high expression in other body parts, with *AHA39270.1*_D1 showing high expression in the forelegs during the feeding stage of female beetles. *evm.TU.chr*2.1058, *XP_015836450.1*_D1, *evm.TU.chr*3.1558, and e*vm.TU.chr*8.525 demonstrated high expression in the antennae. *evm.TU.chr*2.1058 was exclusively high in expression in the mouthparts and antennae, while e*vm.TU.chr*3.1558 was exclusively high in expression in the facial region and antennae. *evm.TU.chr*1.1432, *evm.TU.chr*9.569, *evm.TU.chr*1.1441, *evm.TU.chr*1.1443, *evm.TU.chr*1.1434, *evm.TU.chr*1.1442, and *evm.TU.chr*1.1433 exhibited high expression in the mouthparts. Moreover, *evm.TU.chr*2.211 exhibited high expression only in the antennae during the mating stage of male beetles.

### 2.4. The Analysis of the Spatiotemporal Expression Levels of the OR Genes of M. alternatus

**Figure 4:**
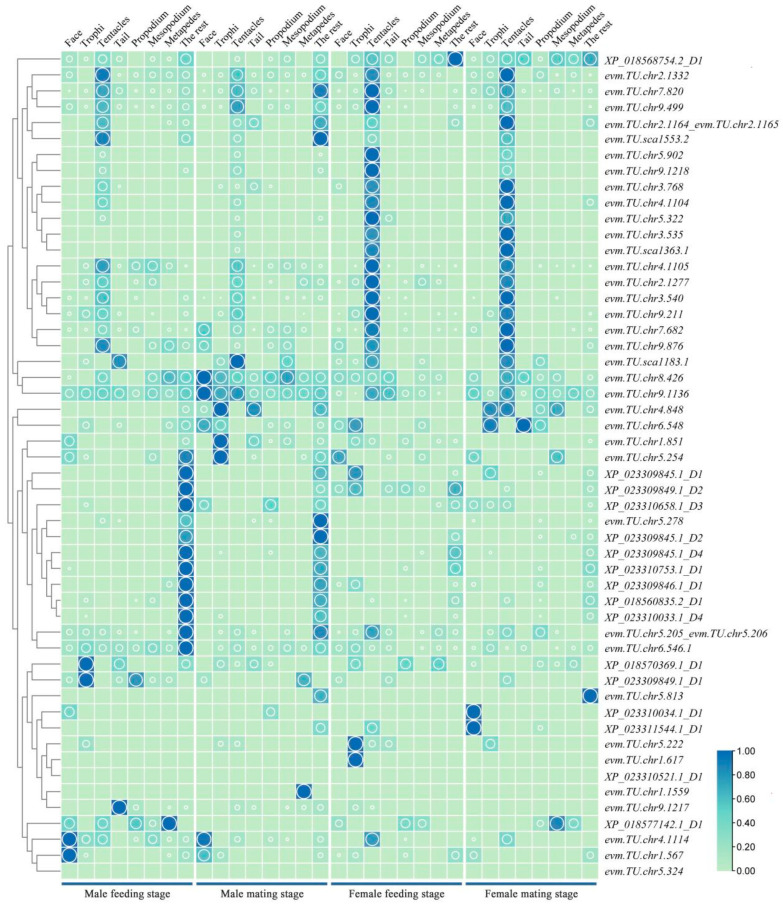
Spatiotemporal expression heatmap of OR genes in *M. alternatus*. Green represents the low expression level of OR genes in various tissues of *M. alternatus*, blue represents the high expression level of OR genes in various tissues of *M. alternatus*. The larger the circle, the higher the expression level.

Based on the genomic data of *M. alternatus*, the spatiotemporal expression profiles of 52 OR genes were established (Figure 4). The OR genes are primarily concentrated in the antennae of female beetles and other body parts of male beetles, with 13 genes exhibiting high expression in the antennae and 11 genes exhibiting high expression in other body parts. Among them, *evm.TU.chr*8.426 and *evm.TU.chr*9.1136 were expressed in multiple body parts, with higher expression in the facial region during the mating stage of male beetles and higher expression in the antennae during the mating stage of female beetles. *evm.TU.chr*5.813 was exclusively highly expressed in other body parts during the mating stage. During the feeding stage of male beetles, 11 genes were highly expressed in other body parts, with *XP_081570369.1*_D1 and *XP_023309849.1*_D1 exhibiting high expression in the facial region and *evm.TU.chr*9.1217 and *evm.TU.sca*1183.1 exhibiting specifically high expression in the antennae. During the mating stage of male beetles, six genes were highly expressed in other body parts, three genes exhibited high expression in the mouthparts, three genes exhibited high expression in the facial region, *evm.TU.sca*1183.1 exhibited high expression in the antennae, and *evm.TU.chr*1.1559 exhibited specifically high expression in the hind legs. During the feeding stage of female beetles, 20 genes were highly expressed in the antennae, *XP_018568754.2*_D1 was highly expressed in other body parts, and *evm.TU.chr*5.222 and *evm.TU.ch*1.617 exhibited high expression in the mouthparts, with *evm.TU.ch*1.617 being specifically highly expressed in female beetles during this stage. During the mating stage of female beetles, 15 genes were highly expressed in the antennae, while *XP_023310034.1*_D1 and *XP_023311544.1*_D1 exhibited specifically high expression in the facial region.

### 2.5. The Analysis of the Spatiotemporal Expression Levels of the GR Genes of M. alternatus

**Figure 5:**
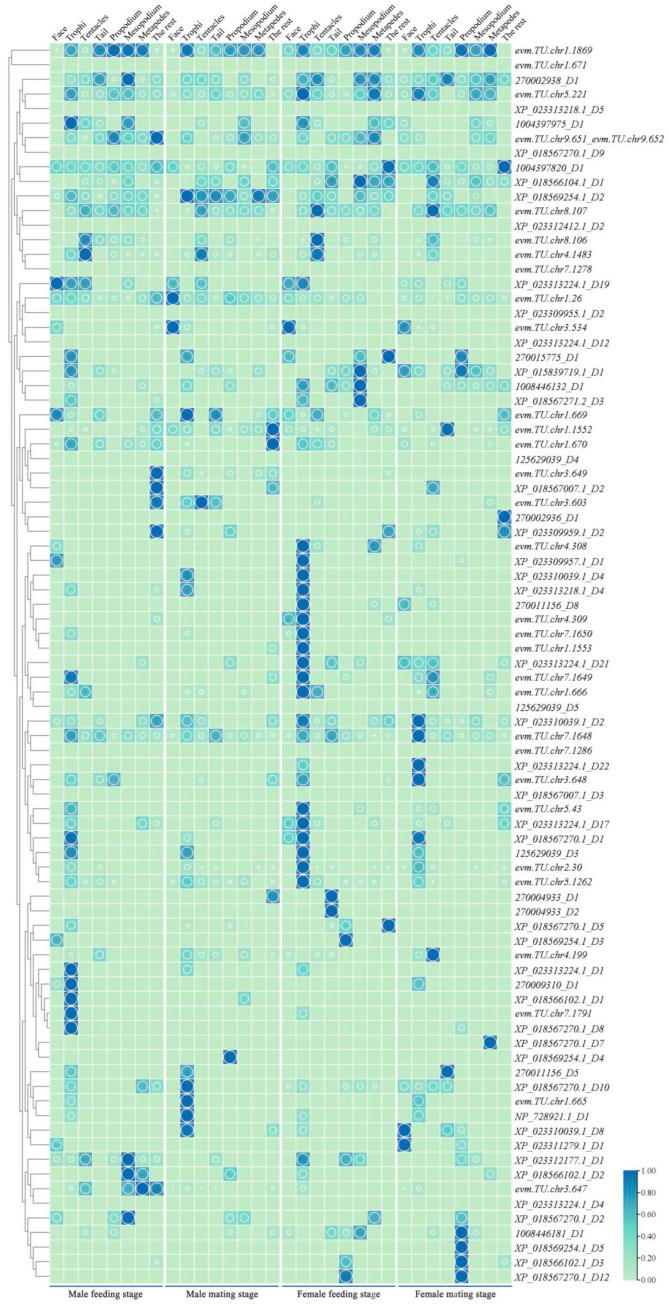
Heatmap of GR genes expression in different parts of *M. alternatus*. Green represents the low expression level of GR genes in various tissues of *M. alternatus*, blue represents the high expression level of GR genes in various tissues of *M. alternatus*. The larger the circle, the higher the expression level.

The results indicated that the expression levels of GR genes were low, with the main expression enrichment occurring in the mouthparts. Among them, genes such as *evm.TU.chr*1.1869, *270002938*_D1 and *evm.TU.chr*5.221 were expressed in all body parts (Figure 5).

During the feeding stage of male beetles, 44 genes were expressed in the mouthparts, with 14 genes showing specifically high expression only in the mouthparts. Among them, *evm.TU.chr*4.308, *XP_023309957.1*_D1, *XP_017569254.1*_D3, and *XP_023312177.1*_D1 exhibited specifically high expression in the facial region, while *evm.TU.chr*3.649, *XP_018567007.1*_D2, *evm.TU.chr*3.603, and *XP_02330957.1*_D2 exhibited specifically high expression in other body parts. During the mating stage of male beetles, 36 genes were expressed in the mouthparts, with 11 genes showing specifically high expression only in the mouthparts. Additionally, four genes exhibited specific expression only in the forelegs, and four genes exhibited specific expression in other body parts. Moreover, *XP_023312177.1*_D1 and *XP_023313224.1*_D17 were exclusively expressed in the tail region.

During the feeding stage of female beetles, 47 genes were expressed in the mouthparts, with 11 genes exhibiting specifically high expression only in the mouthparts. *XP_018567270.1*_D12, *XP_017566102.1*_D3, and *XP_018569254.1*_D3 exhibited specific expression in the forelegs, *XP_018567270.1*_D2 exhibited specific expression in the hind legs, *evm.TU.chr*3.649 and *XP_023309959.1*_D2 demonstrated specific expression in other body parts, *evm.TU.chr*3.603 exhibited specific expression in the antennae, and *evm.TU.chr*8.107, *270002938*_D1, and *evm.TU.chr*4.1483 were highly expressed in the antennae. During the mating stage of female beetles, 35 genes were expressed in the mouthparts, with seven genes showing specifically high expression only in the mouthparts. *evm.TU.chr*1.1552, *270002938*_D1, and *270011156*_D5 exhibited high expression in the tail region, while three genes exhibited specific expression in the hind legs, and five genes demonstrated specific expression in the forelegs. *1004397820*_D2, *270002936*_D1, and *XP_023309959.1*_D2 were highly expressed in other body parts.

### 2.6. The Analysis of the Coexpression of the IR Genes of M. alternatus

**Figure 6:**
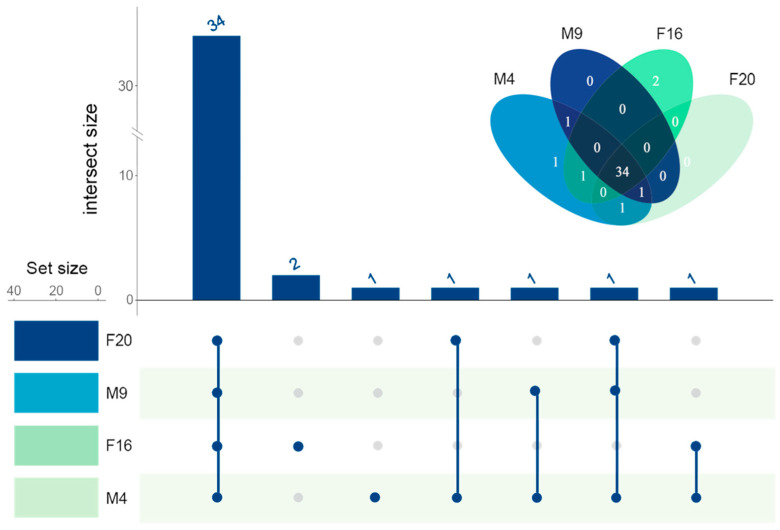
IR gene co-expression map of *M. alternatus* at different ages. M4: male feeding stage; M9: male mating stage; F16: female feeding stage; F20: female feeding stage.

Based on the expression levels of *M. alternatus* across the four developmental stages, a co-expression analysis revealed that there were 34 genes expressed in all four stages, as illustrated in Figure 6. Additionally, in the feeding stage of female beetles (F16), two genes were uniquely expressed, namely *MalIR5* and *MalIR39*; in the feeding stage of male beetles (M4), the gene specifically expressed is *MalIR34*; the gene expressed only in both developmental stages of male beetles is *MalIR37*; the gene expressed only in the feeding stage of female beetles (F16) and male beetles (M4) is *MalIR33*; the gene expressed only in the feeding stage of male beetles (M4) and the mating stage of female beetles (F20) is *MalIR36*; the gene expressed only in both developmental stages of male beetles and the mating stage of female beetles (F20) is *MalIR8*. The expression levels of these uniquely expressed genes are relatively low, primarily distributed in the AMPA subfamily, with *MalIR8* being the only gene in the Antennal IR subfamily.

### 2.7. The Analysis of the Spatiotemporal Expression Levels of the IR Genes of M. alternatus

**Figure 7:**
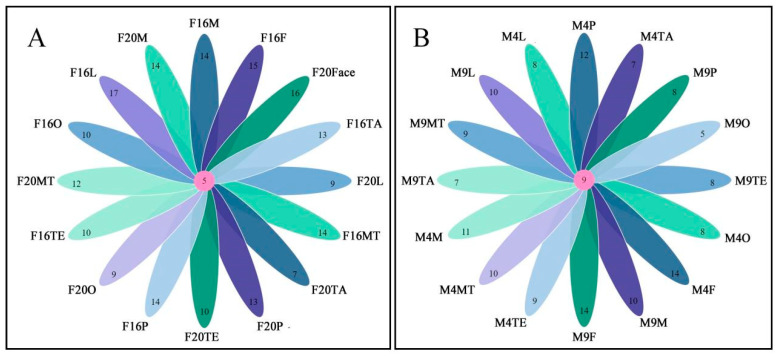
IR gene co-expression map of *M. alternatus* in different parts. (**A**) Co-expression map of IR genes in different parts of females; (**B**) co-expression map of IR genes in different parts of males.

A co-expression analysis was conducted on the IR gene expression, as shown in Figure 7. The results indicated that in female beetles, *MalIR17*, *MalIR22*, *MalIR25*, *MalIR41*, and *MalIR45* were expressed in all body parts. The highest number of genes expressed in the feeding stage was 17 in the mouthparts, followed by 15 in the facial region; 14 each in the forelegs, middle legs, and hind legs; 13 in the tail region; and 10 genes showing high expression in the antennae and other body parts. In the mating stage, the highest number of genes expressed in the facial region was 16, followed by nine in the mouthparts and other body parts; 13, 14, and 12 in the forelegs, middle legs, and hind legs, respectively; seven in the tail region; and 10 in the antennae (Figure 7A).

In male beetles, *MalIR11*, *MalIR15*, *MalIR17*, *MalIR22*, *MalIR24*, *MalIR25*, *MalIR41*, *MalIR42*, and *MalIR44* were expressed in all body parts. In the feeding stage, the highest number of genes expressed in the facial region was 14, followed by 12 in the forelegs, 11 in the middle legs, and 10 in the hind legs. There were nine genes expressed in the antennae, seven in the tail region, and eight genes in each of the mouthparts and other body parts. In the mating stage, the highest number of genes expressed in the facial region was 14, followed by eight in the forelegs, ten in the middle legs, and nine in the hind legs. There were eight genes expressed in the antennae, seven in the tail region, ten in the mouthparts, and five genes in the other body parts (Figure 7B).

**Figure 8:**
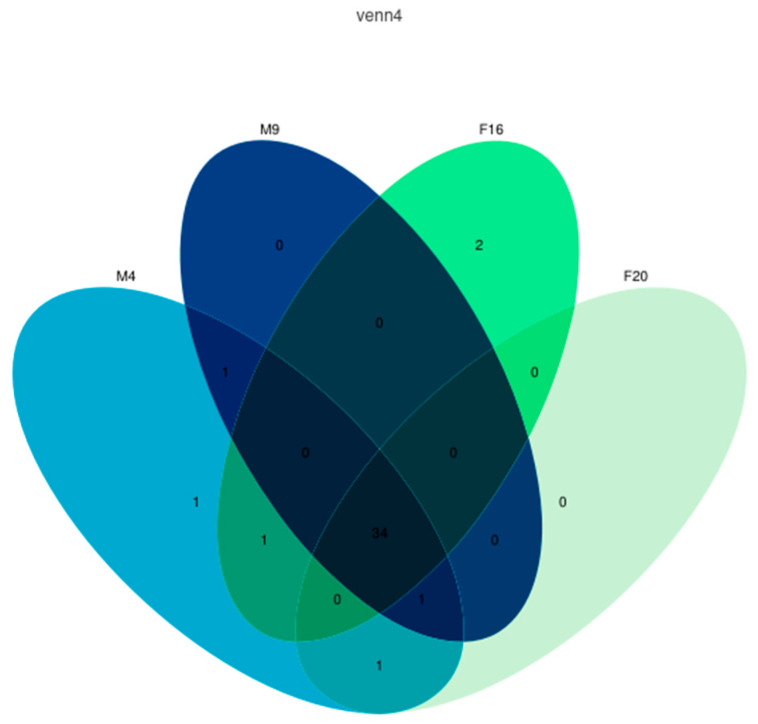
Co-expression map of IR genes at different stages in male and female *M. Alternatus*.

Based on transcriptomic data, a co-expression analysis of genes expressed in the antennae of *M. alternatus* revealed that 30 IR genes were expressed in all stages, as shown in Figure 8. Specifically, *MalIR7* and *MalIR30* were only expressed during the feeding stage of female beetles; *MalIR16* was co-expressed across different stages in male beetles and during the feeding stage in female beetles; *MalIR20* was co-expressed during the mating stage in male beetles and the feeding stage in female beetles; *MalIR8* was co-expressed across different stages in male beetles and during the mating stage in female beetles.

**Figure 9:**
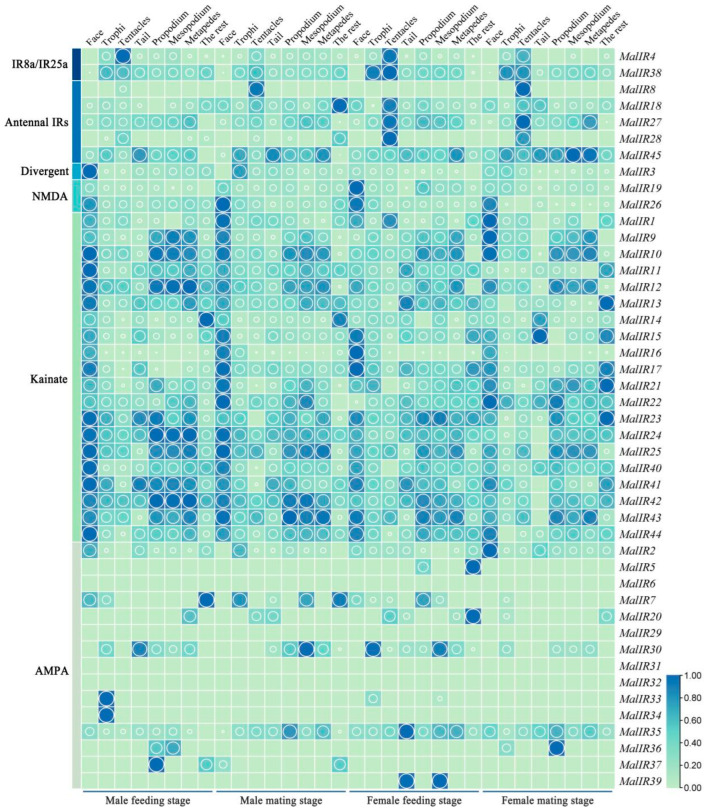
Spatiotemporal expression heatmap of IR genes in *M. alternatus*. Green represents the low expression level of IR genes in various tissues of *M. alternatus*, blue represents the high expression level of IR genes in various tissues of *M. alternatus*. The larger the circle, the higher the expression level.

45 IR genes were established using a heatmap based on the genomic data of *M. alternatus* (Figure 8). The results indicated that the expression levels of IR genes in *M. alternatus* were relatively high, with a large number of genes being expressed. Among them, the Antennal IR and IR8aIR25a subfamily genes exhibited higher expression in the antennae, with female expression levels higher than males. Specifically, *MalIR8* exhibited specifically high expression in the antennae during the mating stage, *MalIR4* exhibited high expression in the antennae during the feeding stage, *MalIR28* exhibited high expression in the antennae of female beetles, and *MalIR45* was expressed in all body parts, with particularly high expression levels in the tail and legs. The divergent subfamily *MalIR3* was predominantly expressed in the facial region, antennae, and mouthparts, with high expression in the facial region during the feeding stage in male beetles. The NMDA and Kainate subfamily genes were expressed in the facial region, as well as in the antennae and legs, with *MalIR16* exhibiting high expression in the facial region. The AMPA subfamily genes had lower expression levels, with *MalIR34* and *MalIR33* showing high expression in the mouthparts during the feeding stage in male beetles; *MalIR34* was exclusively expressed in the mouthparts, *MalIR39* exhibited specifically high expression in the tail and middle legs during the feeding stage of female beetles, and *MalIR5* exhibited expression in the forelegs and other body parts during the feeding stage of female beetles (Figure 9).

## 3. Discussion

Insects rely on chemical cues to recognize important volatile signals, which are crucial for behaviors such as foraging, oviposition, mating, or evading predators. Therefore, the reception of chemical information is indispensable for the survival and life processes of animals [18]. Furthermore, the role of chemical signal recognition may also contribute to species evolution, such as reproductive isolation and speciation. The chemosensory receptors that play a key role in the biological processes of organisms serve as a starting point for studying the role of natural selection in molecular adaptation.

Insects’ IRs are widely distributed throughout their bodies, including sensory organs in the labellum, legs, pharynx, and wings, playing a role not only in gustatory responses but also in oviposition and courtship behaviors [19,20]. In some neurons, IRs and GRs are co-expressed [20]. Despite originating from different gene families, different chemosensory receptors may also exhibit co-expression. For instance, the Orco gene in the OR gene family and the *IR25a* gene in the IR gene family are extensively co-expressed [21]. Building upon the identification of 44 OBP genes, 52 OR genes, 85 GR genes, 15 CSP genes, 45 IR genes, and three SNMP genes in the *M. alternatus* genome, further analyses of their spatiotemporal expression patterns within the beetle are essential.

CSPs constitute a highly expressed soluble small peptide family in insect chemosensory organs with high levels of expression in the chemosensory lymph. They play a crucial role in chemical communication and recognition but also have functions beyond recognition, such as in development and insecticide resistance [22]. In Orthopteran species *Locusta migratoria* and *Schistocerca gregaria*, CSPs are mainly classified into CSP-I and CSP-II. Phenylacetonitrile exhibits good affinity with CSP-II but not with CSP-I, and this compound is considered a component of the pheromone of desert locusts [23]. Studies on the parasitoid wasp *Encarsia formosa* have shown that CSP-III can bind to a variety of host volatiles, such as dimethyl phthalate, 1-octene, β-ocimene, and terpenes [24]. CSPs show a relatively high expression level in *M. alternatus*, with no significant difference in expression between male and female beetles. They are expressed in various body parts, with *evm.molel.chr*2.1459, *evm.molel.chr*2.1457, and *evm.molel.chr*2.1460 showing high expression in the antennae, indicating their potential role as key genes in recognizing informational compounds in *M. alternatus*. *evm.molel.chr*2.1451 and *evm.molel.chr*2.1455 exhibit high expression in the mouthparts, suggesting their possible involvement in the feeding and oviposition site selection behaviors of *M. alternatus*.

SNMPs are insect-specific membrane proteins initially discovered in olfactory sensory neurons (OSNs) sensitive to pheromones in Lepidoptera [25,26,27]. They act as co-receptors and interact with various OBPs and ORs in odor detection processes. They are mainly classified into SNMP1s, SNMP2s, and SNMP3s, serving as key players in insect pheromone recognition. Meanwhile, SNMP4s, as a novel type of SNMP gene, are distributed in the tissues of Coleoptera. SNMP genes are fewer in number, yet they exhibit relatively high expression levels in *M. alternatus*, with no significant difference in expression between male and female beetles. They show high expression in the antennae, mouthparts, and other body parts, with *evm.molel.chr*5.351 showing high expression in other body parts (thorax, abdomen, wings, etc.), potentially serving as a key gene in recognizing informational compounds. Moreover, *evm.molel.chr*5.352 exhibits high expression in the mouthparts, suggesting its possible importance in the feeding behavior of *M. alternatus*.

The insect OBP gene encodes odor-binding proteins, which help transmit odor molecules to sensory neurons. The *Drosophila melanogaster* and fruit fly OBP57d and OBP57e play a role in host selection [28]. The OBP13 of *M. alternatus* showed specificity in the recognition of 20 host odors, with 4-ethylphenol identified as the optimal ligand with a KD value of 0.77 μM [29]. In our reaserch, the expression level of OBPs is relatively high in *M. alternatus*, and multiple MalOBPs are expressed in the mouthparts, antennae, tail, and legs. The expression level of MalOBPs is higher in female beetles compared to male beetles. The presence of MalOBPs in the mouthparts and legs suggests that these genes may mediate behaviors such as foraging and mating in *M. alternatus*. For instance, in the research studies on *Cylas formicarius*, OBP4-6 has been shown to have a strong binding affinity with ligands such as sex pheromones [22]. Genes like *evm.TU.chr*2.211, *evm.TU.chr*8.525, and *evm.TU.chr*3.1558 exhibit specifically high expression in the antennae of female beetles, indicating their potential as key genes for recognizing informational compounds in *M. alternatus*. Genes such as *evm.TU.chr*1.1432, *evm.TU.chr*9.569, *evm.TU.chr*1.441, and *evm.TU.chr*1.443 show specifically high expression in the mouthparts, which suggests their possible involvement in the feeding behavior of the beetle.

The OR gene of insects encodes odor receptors, which are involved in the signal transduction of odor perception. Their main function is to bind odor molecules and transmit signals to downstream proteins (G proteins). The mechanism in insects differs from that in vertebrates regarding olfactory receptors and metabolism [30,31,32]. In the research on the Dipteran species *Bactrocera dorsalis* Hendel, it was shown that OR24 and Orco exhibit significant responses to linalool [33]. ORs exhibit a lower expression level in *M. alternatus*, with most MalOR genes showing specifically high expression in the antennae, and the expression level is higher in female beetles compared to male beetles, consistent with the expression pattern of OBPs. Genes such as *evm.TU.chr*8.426, *evm.TU.chr*9.1136, *evm.TU.chr*4.1114, and *evm.TU.chr*1.567 are highly expressed in the facial region of female beetles, suggesting their potential importance in the feeding behavior of *M. alternatus*. Genes like *evm.TU.chr*9.1217, *evm.TU.chr*6.548, and *evm.TU.chr*4.848 show high expression in the tail region of female beetles, indicating their potential role as key genes in the mating behavior of *M. alternatus*. In male beetles, only *evm.TU.chr*5.813 is highly expressed in other body parts (thorax, abdomen, wings, etc.), suggesting its potential role as a key gene in recognizing informational compounds. Moreover, *evm.TU.chr*1.1559 exhibits specifically high expression only in the hind legs during the mating stage of male beetles, indicating its potential importance in sexual behavior or oviposition in *M. alternatus*.

The insect GR gene is an important component of the insect taste system, responsible for sensing and transmitting signals of food taste. These receptors are widely distributed on insect mouthparts, helping insects identify potential food sources and toxic substances [34]. In the study of bitter taste function in the Dipteran species *Drosophila sechellia*, it was also shown to be the core taste receptor for bitter compounds [35]. Chemoreceptors located on the labellum and tarsi detect sweet compounds, thereby activating proboscis extension and feeding behavior [36]. GRs demonstrate a lower expression level in *M. alternatus*, mainly due to their function in gustatory recognition. Therefore, most MalGR genes show specifically high expression in the mouthparts, with a higher expression level in female beetles compared to male beetles, consistent with the expression patterns of OBPs and ORs. During the mating stage of male and female beetles, genes exhibiting specifically high expression in the tail region include *evm.TU.chr*1.1552, *270002938*_D1, *270011156*_D5, *XP_023312177.1*_D1, and *XP_023313224.1*_D17, indicating their potential role as key genes in sexual behavior or oviposition in *M. alternatus*.

The insect IR gene encodes an Ionic Receptor, which is a key molecule for insects to perceive internal stimuli such as temperature. These receptors play a role in insect sensory organs, helping insects perceive and adapt to temperature changes in the environment [37]. The number and composition of insect IR gene families may vary significantly among different insect species. Some species may have fewer IR genes, while others may have more IR genes. This diversity reflects the differences in the recognition and perception needs of chemical substances among different insect species. In the Lepidopteran species *Helicoverpa armigera*, A-IRs show higher expression levels in the antennae of adults or larvae than in other tissues, and they are also detected in the proboscis and legs, suggesting that some A-IRs in *H. armigera* may have dual functions in both olfaction and taste [38]. IRs exhibit stable and high expression levels in *M. alternatus*, with 34 genes being co-expressed in both male and female beetles across two stages. Most MalIR genes show specifically high expression in the facial region and antennae, with 30 genes co-expressed in the antennae at various stages. The expression levels in male beetles are higher than in female beetles, indicating the involvement of IR genes in the recognition processes of *M. alternatus*. The Antennal IR and IR8a/IR25a subfamilies exhibit high expression in the antennae, while the Kainate subfamily shows lower expression levels in the antennae, with expression mainly concentrated in the facial region. Genes such as *MalIR1*, *MalIR4*, *MalIR38*, *MalIR18*, *MalIR27*, and *MalIR28* are highly expressed in the antennae during the feeding stage of female beetles, suggesting their important role in recognition during this stage. *MalIR8* exhibits high expression in the antennae during the mating stage of both male and female beetles, indicating its key role in the mating behavior of *M. alternatus*. Genes like *MalIR45*, *MalIR24*, and *MalIR42* are expressed across all stages, indicating potential roles in multiple recognition functions. *MalIR3* shows a specifically high expression in the facial region of male beetles.

## 4. Materials and Methods

### 4.1. The Test Insect

The test insect is a strain of *M. alternatus* bred in our laboratory. The rearing conditions are as follows: rearing temperature—23–26 °C; light–dark cycle—L:D = 12:12; relative humidity—70–75%.

**Figure 10:**
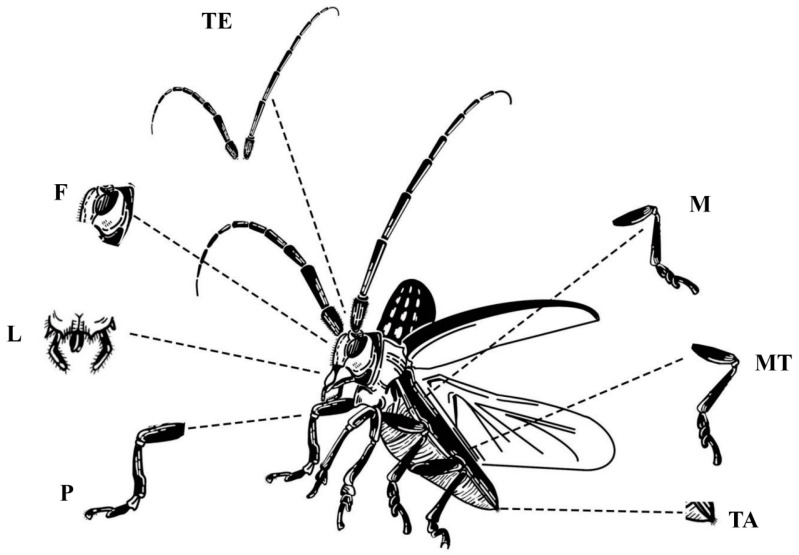
Transcriptome sequencing samples of IR genes in *M. alternatus*. F: face; L: lips; TE: tentacles; TA: tail; P: propodium; M: mesopodium; MT: metapedes.

Transcriptome sequencing samples: Samples were collected from different developmental stages, genders, and body parts of *M. alternatus*, with 3 replicates per group (Table 1). Each replicate is a mixture of 5 samples. Due to the different growth conditions of individual insects, there may be a one-week gap between the growth of individual insects. After dissection, the samples were promptly labeled, placed in 1.5 mL centrifuge tubes, and stored at −80 °C for future use, as shown in Figure 10.

### 4.2. RNA Isolation and Illumina Sequencing

Total RNA was extracted by Trizol reagent (Invitrogen, Carlsbad, CA, USA). Amounts of 50–100 mg tissues were added to 1.5 mL Trizol, vortexed, and incubated at room temperature for 5 min to ensure complete lysis. They were centrifuged at 12,000× *g* for 5 min and then the supernatant was transferred to a new tube. Chloroform was added at a ratio of Trizol:chloroform = 5:1, the tube was covered tightly, vortex-mixed for 15 s in a vortex shaker, and incubated at room temperature for 2–3 min to allow for phase separation. The sample was centrifuged at 4 °C, 12,000× *g* for 10 min. The aqueous phase was then transferred to a new tube, an equal volume of chloroform was added to the tube, it was vortex-mixed for 15 s in a vortex shaker, incubated at room temperature for 2–3 min to allow for phase separation, and centrifuged at 4 °C, 12,000× *g* for 10 min. The upper aqueous phase was then transferred to a new 1.5 mL EP tube, an equal volume of isopropanol was added to the tube, and it was incubated at room temperature for 10 min. The sample was centrifuged at 4 °C, 12,000× *g* for 15 min, then the supernatant was discarded. Then, 1 mL 75% ethanol was added to the RNA pellet, vortex-mixed for 5 s in a vortex shaker, centrifuged at 4 °C, 7500× *g* for 5 min, and then the supernatant was discarded. The RNA pellet was placed in a sterile workbench to air dry for approximately 5–10 min, and then the RNA pellet was dissolved by DEPC-treated water.

The purity of RNA was determined by the Nanodrop spectrophotometer (IMPLEN, Westlake Village, CA, USA) and detection, and the integrity was accurately determined by RNA Nano 6000 Assay Kit of the Bioanalyzer 2100 system (Agilent Technologies, Santa Clara, CA, USA). Sequencing libraries were generated using NEBNext^®^Ultra^TM^ RNA Library Prep Kit for Illumina^®^ (NEB, Ipswich, MA, USA). Oligo(dT) magnetic beads were used to enrich mRNA with polyA tails. Subsequently, the first cDNA strand was synthesized and subjected to PCR amplification. Finally, the PCR products were purified to obtain the library required for this study. After constructing the sequencing library, the library was subjected to initial quantitative analysis using a Qubit 2.0 Fluorometer. Upon confirming the library’s quality through this detection, both the insert size and effective concentration of the library were measured. Further precise quantitative analyses using qRT-PCR were conducted to ensure the quality of the library. Then, the high-throughput RNA-sequencing libraries were prepared, following Illumina’s protocols, and were sequenced on the Illumina NovaSeq 6000 sequencing platform (Illumina, SanDiego, CA, USA).

### 4.3. De Novo Transcriptome Assembly and Annotation

The raw reads were filtered to remove adaptor fragments, i.e., reads containing unknown nucleotide “N” over 5% and empty tags to obtain high-quality clean reads. The fastp software (version 0.23.1) was used to conduct data quality control. The TGICL v2.1 clustering tool was utilized to assemble all the unigenes with default parameters [39]. Then, the unigenes were aligned with the Nr, Swiss-Prot, COG, KOG, and eggNOG4.5 databases by BLAST v2.2.31 [40], and GO annotation was performed using Blast2GOv2.5. The HMMER v3.1b2 software was used to search the Pfam database to obtain the annotation information of the unigenes.

### 4.4. Differential Expression Analysis of Chemosensory Receptor Protein Genes

The UpSet image from NovoMagic was used to analyze differential expression (https://magic.novogene.com accessed on 1 March 2024), and the parameter was the fpkm value. To investigate the functional characteristics of the chemosensory receptor protein gene family in supplemental nutrition and the mating oviposition stages of *M. alternatus*, the FPKM values of the chemosensory receptor protein gene family were extracted based on the transcriptome database for the feeding stages (male eclosion at 4 days (M4) and female eclosion at 16 days (F16)) and mating stages (male eclosion at 9 days (M9) and female eclosion at 20 days (F20)) [41]. The spatial and temporal expression patterns of chemosensory receptor protein genes in *M. alternatus* were visualized using the HeatMap tool in the TBtools software v2.07 [42]. The calculation process of FPKM values is as follows:

$${\mathrm{FPKM}}_{i}=\frac{X_{i}}{\left(\frac{\tilde{l_{i}}}{10^{3}}\right)\left(\frac{N}{10^{6}}\right)}=\frac{X_{i}}{{\tilde{l}}_{i}N}\cdot 10^{9}$$

In the formula, *N* represents the total number of sequenced reads, *X_i_* represents the count number of genes, and *l* represents the length of the gene (measured in base pairs) [43].

## 5. Conclusions

In conclusion, the receptors in the olfactory sensory system operate independently at the molecular level and are also subject to regulation during various developmental processes, each located in distinct structures of receptors. Therefore, the chemosensory receptors mentioned above appear to have distinct functions in chemical compensation. Building upon this foundation, it can be demonstrated that elucidating the functions of the aforementioned genes is crucial for a better understanding of the essential interplay pathways between different insect olfactory receptors and host plants. This study provides a theoretical basis for the development of novel attractants or repellents for *M. alternatus*, thus playing a significant role in curbing the spread of pine wilt disease.

## Figures and Tables

**Table 1:** The neurotranscriptome sequencing samples of *M. alternatus.*

Gender	Instar	Sampling Location
Male	4 d post-eclosion (feeding stage)	Face Trophi Tentacles Tail Propodium Mesopodium Metapedes The rest
	9 d post-eclosion (mating stage)	
Female	16 d post-eclosion (feeding stage)	
	20 d post-eclosion (mating stage)	

## Data Availability

Data are contained within the article.
